# Characterization of Stripe Rust Resistance Genes in the Wheat Cultivar Chuanmai45

**DOI:** 10.3390/ijms17040601

**Published:** 2016-04-21

**Authors:** Ennian Yang, Guangrong Li, Liping Li, Zhenyu Zhang, Wuyun Yang, Yunliang Peng, Yongqing Zhu, Zujun Yang, Garry M. Rosewarne

**Affiliations:** 1 Crop Research Institute, Sichuan Academy of Agricultural Sciences, Chengdu 610066, China; anzerna@126.com (L.L.); yangwuyun@126.com (W.Y.); garry.rosewarne@ecodev.vic.gov.au (G.M.R.); 2 School of Life Science and Technology, University of Electronic Science and Technology of China, Chengdu 610054, China; ligr28@uestc.edu.cn (G.L.); yangzujun@uestc.edu.cn (Z.Y.); 3 Institute of Plant Protection, Sichuan Academy of Agricultural Sciences, Chengdu 610066, China; zhzhyu1982@163.com (Z.Z.); pengyunliang@yahoo.com (Y.P.); 4 Institute of Agro Products Processing Science and Technology, Sichuan Academy of Agricultural Sciences, Chengdu 610066, China; zhuyongqing68@sina.com; 5 International Maize and Wheat Improvement Centre (CIMMYT), Apdo. Postal 6-6-41, Mexico 06600, D.F., Mexico; 6 Department of Environment and Primary Industries, 110 Natimuk Rd, Horsham, Victoria 3401, Australia

**Keywords:** stripe rust resistance, wheat-rye 1RS.1BL translocations, Chuanmai45

## Abstract

The objective of this research was to characterize the high level of resistance to stripe that has been observed in the released wheat cultivar, Chuanmai45. A combination of classic genetic analysis, molecular and cytogenetic methods were used to characterize resistance in an F_2_ population derived from Chuanmai45 and the susceptible Chuanmai42. Inheritance of resistance was shown to be conferred by two genes in Chuanmai45. Fluorescence *in situ* hybridization (FISH) was used along with segregation studies to show that one gene was located on a 1RS.1BL translocation. Molecular markers were employed to show that the other locus was located on chromosome 4B. The defeated gene, *Yr24/26*, on chromosome 1BL was present in the susceptible parent and lines that recombined this gene with the 1RS.1BL translocation were identified. The germplasm, loci, and associated markers identified in this study will be useful for application in breeding programs utilizing marker-assisted selection.

## 1. Introduction

Wheat stripe rust (yellow rust), caused by *Puccinia* *striiformis* (PST) Westend. f. sp. *tritici* Eriks., is a major disease of wheat (*Triticum* *aestivum* L.) worldwide, and can cause yield losses as high as 75% in extremely susceptible cultivars [1]. Growing resistant cultivars is the most economic, effective and environment-friendly approach to control the disease [2]. To date, more than 70 stripe rust resistance genes with official and provisional designations have been reported in wheat [3]. The majority of these genes are race-specific with virulence being detected in various parts of the world, rendering cultivars carrying these genes susceptible [4]. In China, there are very few major genes that are still effective to all pathotypes. It is, therefore, urgent to identify new effective genes for resistance to stripe rust and to pyramid the different genes into wheat cultivars.

Chen *et al.* [5] analyzed the stripe rust pathogen populations in China and reported that a few races, namely CYR32, CYR33, Su11-4, and Su11-7, have predominated in areas prone to stripe rust. Among the officially-named stripe rust resistance genes, only *Yr5*, *Yr10*, *Yr15*, and *Yr24/26* confer resistance to the race CYR32 [5,6,7]. Cultivars with *Yr24/26* have been rapidly released in recent years [8]. However, a new virulent stripe rust race, Accession No. 09-6-16-3, also termed v26, is virulent to this locus [9] and this pathotype is increasing in frequency. It is, therefore, essential to identify new stripe rust resistance genes that are effective against the *Yr26* virulent race. Molecular markers associated with the new resistance genes are also desirable as they can facilitate the rapid deployment of the new genes through marker-assisted selection (MAS).

Chuanmai45 (CH45), a spring wheat cultivar developed by the Crop Research Institute, Sichuan Academy of Agricultural Sciences, has high yield potential and was released in 2004. It displayed high resistance to stripe rust in experimental plots in both seedlings and adult plants. In the present study, molecular and cytogenetic analyses were used to characterize the stripe rust resistance in CH45, along with further characterization of the defeated *Yr24/26* gene, carried by Chuanmai42 (CH42) [10,11]. The concomitant identification of molecular markers should be useful for maker assisted selection (MAS) of stripe rust resistance in common wheat backgrounds.

## 2. Results

### 2.1. Assessment of Stripe Rust Resistance

**Figure 1:**
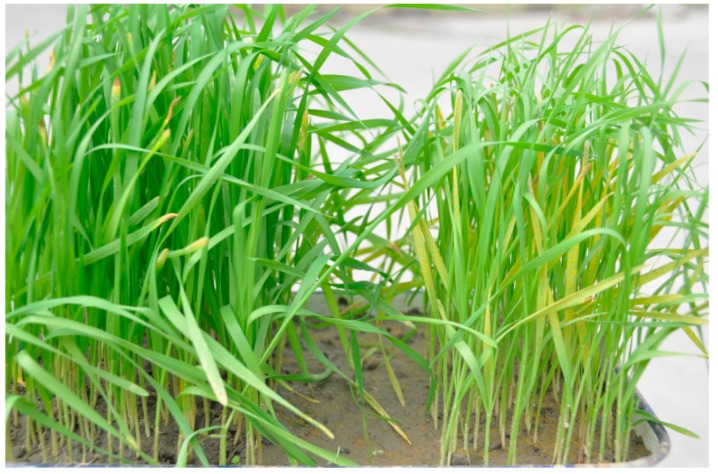
The seedlings of CH45 (**left**) and CH42 (**right**) inoculated by race No. 09-6-16-3.

Reactions of different wheat cultivars or lines with known resistance genes to PST No. 09-6-16-3 were listed in Table 1. The resistance reaction of the wheat lines with known genes were consistent with Liu *et al.* [9]. The lines CN19, containing *Yr41*, and the Avocet NILs, containing *Yr29* and *Yr31*, all showed high susceptibility. CH45 and its parent, SW1862, showed complete resistance to PST No. 09-6-16-3, while CH42 was highly susceptible (Figure 1, Table 1).

### 2.2. Genetic Analysis of Resistance in Chuanmai45 (CH45)

The resistance to stripe rust in the parents and in the F_1_ and F_2_ populations were investigated with infection types (IT) responses of seedlings against v26 (Table 2). The resistant parent (CH45) had an IT of 0, while the two susceptible parents (CH42 and CN19) scored 4. Two F_1_ and F_2_ populations from two crosses, CH45/CH42 and CH45/CN19, were tested. Both F_1_ populations consisted of completely susceptible plants, indicating that the resistance in CH45 was recessive. Furthermore, the F_2_ generation of both CH45/CH42 (192 plants) and CH45/CN19 (206 plants) segregated in a 7:9 ratio for resistance and susceptibility (Table 2). These results are consistent with the presence of two recessive genes, which are hereafter named throughout the text as *yrCH45-1* and *yrCH45-2*.

### 2.3. Bulk Segregate Analysis (BSA) of Resistance

Susceptible and resistant bulks of the CH45/CH42 population were developed by selecting 15 resistant lines (IT0-0;) and 15 susceptible lines (IT4) from the F_2_ population. A total of 168 primer pairs from SSR and expressed sequence tags derived SSR (EST-SSR) primers were screened in the bulk segregate analysis (BSA). There were 40 differentially-amplified fragments between CH42 and the susceptible bulk when compared to CH45 and the resistant bulk (RB). Eight primer pairs (*TOP1017*, *TNAC1009*, *TNAC1021*, *TNAC1045*, *EST123*, *Xgwm11*, *Xgwm18*, and *Xgwm498*) that generated strong and repeatable polymorphic bands had been previously assigned to the 1BS chromosome arm [11,12,13].

### 2.4. In Situ Hybridization of CH45

**Figure 2:**
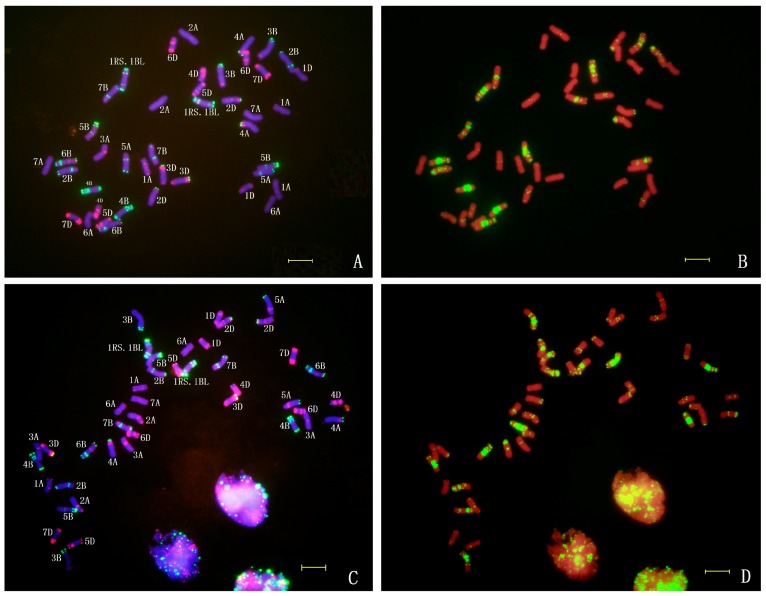
Fluorescence *in situ* hybridization (FISH) of SW1862 (**A**,**B**) and Chuanmai45 (CH45) (**C**,**D**) with (**A**,**C**) stained by DAPI (blue), Oligo-pTa535 (red), and Oligo-pSc119.2 (green), (**B**,**D**) stained by PI (red) and Oligo-(GAA)_7_ (green).Scale bar indicates 10 μm.

In order to reveal the chromosomal constitution of CH45, we performed sequential, multicolor, fluorescence *in situ* hybridization (FISH) with the probes Oligo-pSc119.2, Oligo-pTa535, and Oligo-(GAA)_7_ to mitotic metaphase cells of CH45 and its parents SW1862 (Figure 2). Based on the FISH standard karyotype of wheat and rye chromosomes [14], we showed that both SW1862 (Figure 2A,B) and CH45 (Figure 2C,D) possessed a rye 1R chromosomal short arm substitution on 1BS, indicating that these lines contain 1RS.1BL translocations. Co-incidentally, all 44 lines that were homozygous for 1RS.1BL were also resistant (Table 3). This data, combined with the BSA, give a strong indication that *yrCH45-1* was located on the 1RS of this translocation.

**Figure 3:**
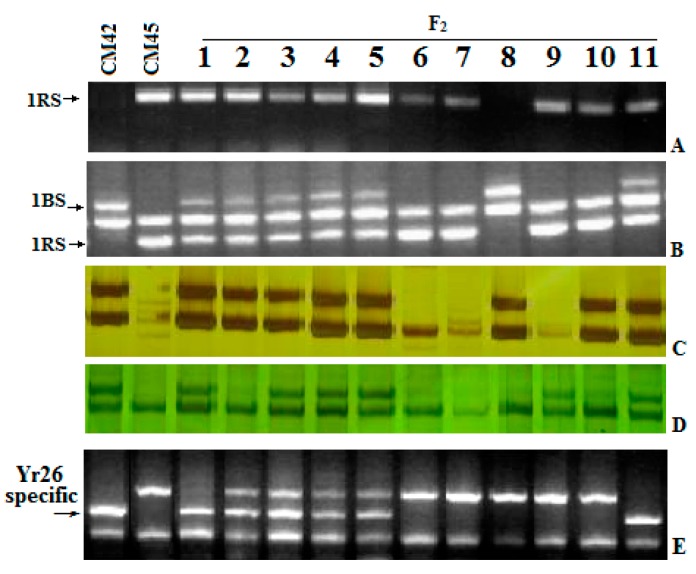
PCR amplification of *Ora12* (**A**); *TOP1017* (**B**); *Xgwm18* (**C**); *Xgwm498* (**D**); and *Xwe173* (**E**) separated by agarose (**A**,**B**,**E**) and silver-stained polyacrylamide gels (**C**,**D**).

**Figure 4:**
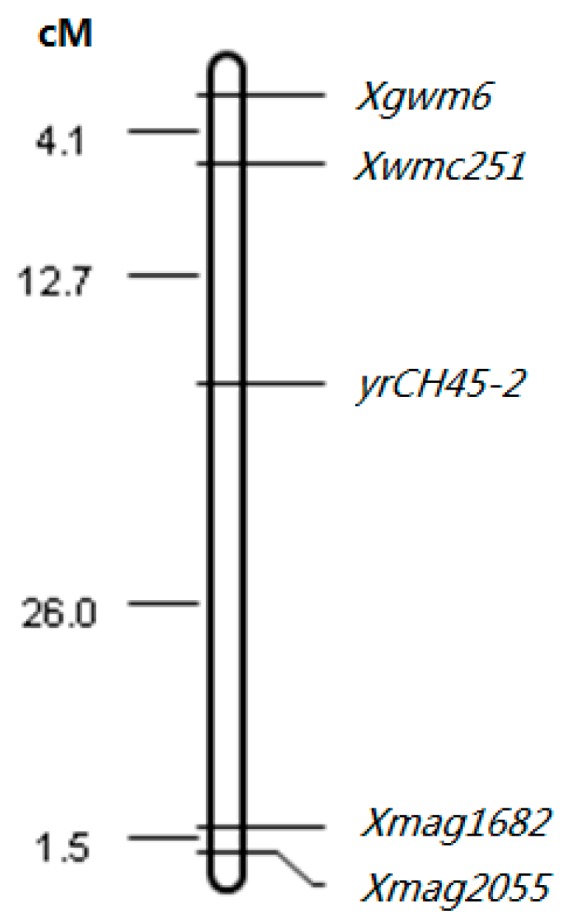
Map position of *yrCH45-2* on chromosome 4BL. Heterozygous F_2_ translocation lines (1RS.1BL) were used to map the resistance locus on 4BL as resistance on the translocation was recessive and would, therefore, not confound phenotypic responses associated with segregation on 4BL.

The F_2_ plants that were 1RS.1BL heterozygotes segregated for resistance in a fashion that indicated the presence of another resistance gene independent of the translocation. This subgroup of translocation heterozygotes segregated in a 1:3 ratio for resistance. This, along with the susceptibility of all F_1_ plants, indicates that this second gene was also recessive in nature. It was temporarily named *yrCH45-2*, and was independent of *yrCH45-1*. A BSA analysis was used to identify molecular markers associated with *yrCH45-2*. A total of 40 markers that differentiated the CH42 and CH45 parental lines were used to screen bulked DNA from lines that segregated for resistance but were heterozygous for the 1RS translocation. Marker *Xwmc251* had a loose association with resistance and this was known to reside on chromosome 4B. A total of 16 more SSR primer pairs and nine EST-SSR markers [12] for chromosome 4B were tested and three more were polymorphic within the populations and in linkage disequilibrium with resistance (Figure 3 and Figure 4). Thus, a recessive gene *yrCH45-2* on 4BL contributed to the rust resistance ITs in CH45. In conclusion, the *yrCH45-1* and *yrCH45-2* were located on 1RS.1BL and 4BL of CH45, respectively.

### 2.5. Recombination of Yr26 in CH45

CH42 contains the *Yr26* gene for stripe rust resistance on chromosome 1B [10,11] and several sequence amplified characterized region (SCAR) markers linked to this gene have been produced [15]. The closest flanking marker, *Xwe173*, is co-dominant and was polymorphic between CH45 and CH42. There were 11 out of the 192 F_2_ plants from the CH45/CH42 population that were homozygous for both *Xwe173* and the 1RS.1BL translocation.

## 3. Discussion

CH45 continues to show high levels of resistance in the face of changing virulence of stripe rust pathogens in China. Pathotypes CYR32 and CYR33 have caused significant disease epidemics in Southwest China in recent years. The evolution of the v26 pathotype is a major concern as it has virulence for *Yr24/26*, a gene that has been widely used throughout China. The combination of two loci may help to increase the durability of CH45. The recessive nature of the resistances identified on a rye translocation and on chromosome 4B have helped to differentiate these loci from other known stripe rust resistances. CH45 also has good yield potential and should make a good parent in crossing programs. The markers identified in this study will facilitate the incorporation of both loci into new germplasm.

The locus *yrCH45-1* was located on a 1RS.1BL translocation. A previous 1RS.1BL translocation from *S. cereale* contained the stripe rust gene *Yr9.* Clearly, the v26 pathogen is virulent on *Yr9*, however, *yrCH45-1* showed an avirulent reaction. Virulence to *Yr9* was indicated by high IT scores on PBW343, Seri82, and Super Kauz (Table 1), all of which contain this gene. The original 1RS.1BL translocation had a significant impact in wheat breeding and global wheat production over several decades. It altered yield components [16] and brought in other resistance genes including *Sr31*, *Lr26*, and *Pm8* [17]. The *Sr31* resistance gene provided stable resistance to stem rust for up to 20 years and was probably the main reason this translocation became so common in global germplasm [4]. However, all resistance genes on this original translocation have now broken down and efforts have been underway to search for different sources of rye chromosome 1RS which contain agronomically useful genes [18].

**Figure 5:**
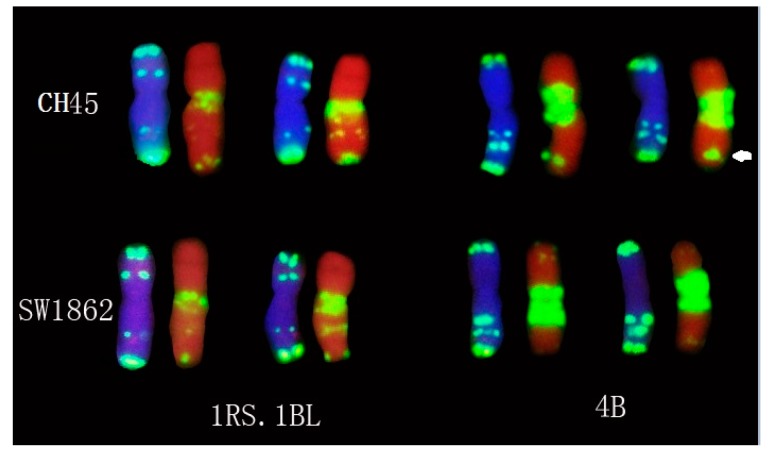
FISH karyotype of chromosomes 1RS.1BL and 4B in SW1862 and CH45. Left chromosomes were stained with DAPI (blue), Oligo-pTa535 (red), and Oligo-pSc119.2 (green), while right chromosomes were stained by PI (red) and Oligo-(GAA)_7_ (green). Arrow shows the unique hybridization on 4B of CH45.

Recently, new 1RS.1BL translocation lines derived from different rye varieties have been developed and will be useful in wheat improvement [19]. Luo *et al.* [20] also developed new 1RS.1BL and characterized two stripe rust resistance loci termed *YrCN17* and *YrR212* originating from the 1RS chromosomal segment. These genes were effective in protecting wheat from the races CYR31 and CYR32. It is likely that *yrCH45-1* is different to *YrCN17* and *YrR212*, as the former locus was inherited in a recessive fashion whereas the latter two are dominant. Based on the FISH hybridization (Figure 2 and Figure 5), it is also likely that *yrCH45-1* is derived from a different source as one of its parents, SW1862,was developed from alternate 1RS.1BL translocations that were distributed by International Maize and Wheat Improvement Centre (CIMMYT) in the 1980s [21]. The germplasm by Luo *et al.* [20] developed much more recently in China and likely used different Rye sources. Next generation sequencing of core 1RS chromosomes should help to characterize diversity of these translocations and facilitate the search for novel resistances for incorporation into bread wheat [22].

There are several other loci on chromosome 1B, including *Yr10*, *Yr15*, *Yr24/26*, *Yr29*, and *YrH52*. The *yrCH45-1* locus is easily differentiated from most of these due to IT data as the pathotype used in this study was virulent on *Yr10* and *Yr24/26* and *Yr29* is an adult plant resistance gene that gives a susceptible IT in seedling tests (Table 1). This CH45 locus can also be differentiated from *Yr15* and *Yr52H* as both of these loci were derived from *T. dicoccoides* translocations onto chromosome 1BS while *yrCH45*-1 has its origins in rye.

The *yrCH45-2* was located on chromosome 4B and may be a new stripe rust resistance gene. Recently, *Yr50* was identified as a dominant gene on chromosome 4BL that was also resistant to the v26 pathotype [23]. Molecular markers place these two loci in similar chromosomal locations; however, the recessive nature of inheritance of *yrCH45-2* gives an indication that it is likely different from *Yr50*. Based on the FISH study of chromosome 4B in CH45 (Figure 2 and Figure 5), we found that the Oligo-(GAA)_7_ gave rise to a strong signal on 4BL of CH45, while SW1862 did not produce such a signal. Furthermore, *Yr50* was introgressed from *Thinopyrum* *intermedium*. There is no information in the pedigree of CH45 that suggests it would have *Th. intermedium* parentage. Allelism tests may not be of use in differentiating these loci as the *Th. intermedium* DNA does not normally recombine with common wheat, and the similar location would render all progeny of such a test as resistant. Genomic *in situ* hybridization (GISH) may be useful in identifying *Th. intermedium* DNA in CH45 and such a result would indicate that *yrCH45-2* could be *Yr50*. The flanking markers of *Xwmc251* and *Xmag1682* were some distance from the locus *yrCH45-2*. Research to identify more tightly-linked markers in breeding programs is underway.

The gene resistance *Yr24* and *Yr26* have been shown to be identical and present in CH42 [10,11]. CH42 was released in 2003 and not only contains *Yr24/26* resistance but has been very widely adopted throughout the region due to its significant yield increase over other existing varieties [24]. Original work identified *Yr26* as being derived from a *T. turgidum* landrace [25] that was used in the creation of the wheat-*Dasypyrum villosum* 6VS/6AL translocation line R64 (92R-149). As the 6VS/6AL translocation also contained the powdery mildew resistance gene, *Pm21* [26], R64 was particularly useful in many wheat-growing regions throughout China. Many Chinese breeding programs have used it to develop new cultivars with powdery mildew and stripe rust resistance [8].

The widespread occupation of *Yr24/26* containing lines has facilitated the growing presence of v26 [9]. Resistance to CYR32 and CYR33 afforded by *Yr24/26* is still useful as these pathotypes remain relatively common in Chinese environments [3]. Closely linked, flanking, co-dominant markers to *Lr24/26* have been identified [15,27]. These provide a useful tool in any marker assisted selection strategy. The 1RS.1BL translocation does not recombine with chromosome 1BS from common wheat and its presence can be determined through the use of the TOP1017 marker. This marker is, therefore, diagnostic for the *yrCH45-1* locus. There were several lines identified in this study that had recombined the 1RS.1BL segment with *Xwe173*, a marker for *Yr24/26*. Given the sound markers available for these loci, it would be relatively easy to maintain this recombination in breeding programs. Such recombinants may be useful in the future should CYR32 or CYR33 develop virulence for the *yrCH45-1* locus. The further pyramiding of the *yrCH45-2* locus from 4B, again with the use of markers, will help develop lines that should be able to retain resistance for a significant period of time.

## 4. Materials and Methods

### 4.1. Plant Materials

CH45 was developed from the cross SW1862/GH430. Cultivars and near-isogenic lines (NIL) with known stripe rust resistance genes in the Avocet background were obtained from Robert A. McIntosh, University of Sydney, Australia. Wheat cultivar CH42 was developed by Wuyun Yang of Sichuan Academy of Agricultural Sciences, and CN19 was developed by Zhenglong Ren of the Sichuan Agricultural University.

### 4.2. Disease Resistance Screening

Pure isolates of the v26 pathotype were used to inoculate CH45, CH42, CN19, SW1862, and their progenies. The avirulence/virulence formula of the new pathotype is *Yr1*, *3*, *4*, *H46*, *5*, *6*, *15*, *17*, *18*, *32*, *Sp*, *Sd/Yr2*, *8*, *9*, *10*, *12*, *24 (=26)*, *31*, *Su* [9].

Seeds were planted in small pots with seven plants of the test line along with three plants of the susceptible cultivar Mingxian 169. Seedlings were inoculated with v26 when the first leaf was fully expanded. After inoculation, the seedlings were placed in a dew chamber at 19 °C and 100% relative humidity for 24 h and then transferred to a greenhouse maintained with 14 h light/10 h dark photoperiod at 12–17 °C. Infection types (IT) were scored 14–15 days after inoculation when rust was fully developed on the susceptible check. Infection types were based on a 0–4 scale [28], with 0 for no visible uredia; 0; for small chlorotic flecks without sporulation; 1 for limited uredial development associated with chlorosis and necrosis; 2 for intermediate sporulation with chlorosis and/or necrosis; 3 for abundant sporulation with chlorosis; and 4 for abundant sporulation without chlorosis. Rating of the seedling reactions was simplified to two classes (resistant and susceptible) as there was a clear distinction between these two categories.

### 4.3. Cytological Analysis

Seedling root tips were collected and then treated with nitrous oxide followed by enzyme digestion, using the procedure of Han *et al.* [29]. The synthesized oligo-nucleotide probes Oligo-pSc119.2, Oligo-pTa535, and Oligo-(GAA)_7_ were used for identifying the wheat chromosomes by FISH according to the description of Tang *et al.* [30]. The protocol of non-denaturing FISH by the synthesized probes was described by Fu *et al.* [12].

### 4.4. Molecular Marker Analysis

Genomic DNA from fresh young leaves was extracted by the SDS method [31] and 2 μL of the DNA solution (*ca.* 100 ng) was used as a template for PCR. Two sets of primers pairs, TOP1017 [32] and Ora12 [33], were used to amplify the 1RS DNA. PCR amplification was carried out in a MyCycleTM Thermal Cycler (Bio-RAD, Hercules, CA, USA). The cycling parameters were 94 °C for 3 min, followed by 40 cycles of 94 °C for 45 s, 55 to 62 °C (depend on the primer) for 45 s, and 72 °C for 2 min, with a final extension at 72 °C for 7 min. An 8 μL aliquot of the product was digested for two hours with 1.0 U each of *Taq*I (65 °C), *Hpa*II (37 °C), and *Hae*III (37 °C). Digested fragments were fractionated by electrophoresis on a 2% agarose gel. The SSR and expressed sequence tags (EST) derived SSR primers and their relevant PCR protocols were described in Xue *et al.* [13].

## 5. Conclusions

This study identified two potentially new resistance genes to stripe rust on chromosome 4B and on a 1RS.1BL rye translocation. These are effective against the v26 pathotype in China. The new genes are inherited in a recessive fashion, a feature that helps to distinguish them from other loci. The translocation was shown to recombine with *Yr24/26*, a 1B locus that has been overcome by v26. Germplasm identified in this study, and markers used here and elsewhere, have the potential to pyramid these loci to provide durable resistance.

## Figures and Tables

**Table 1:** Virulence testing of wheat stripe rust strain pathotype No. 09-6-16-3 on wheat stripe rust differential lines and parental lines at the seedling stage.

Genotypes/Lines	*Yr* Gene Location	Infection Types
Avocet	-	4
Avocet+*Yr1*	2A	0
Kalyansona (*Yr2*, *Yr29*)	7B, 1B	3
Avocet+*Yr5*	2B	0
Avocet+*Yr6*	7B	0
Avocet+*Yr7*	2B	4
Avocet+*Yr8*	2M/2B	4
Avocet+*Yr10*	1B	4
Avocet+*Yr1*5	1B	0
Avocet+*Yr17*, *Yr18*	2A, 7D	3
Avocet+*Yr18*	7D	4
Avocet+*Yr24*	1B	4
Avocet+*Yr26*	1B	4
Avocet+*Yr27*	2B	3
Avocet+*Yr28*	4D	0
Avocet+*Yr29*	1B	4
Avocet+*Yr31*	2B	3
Avocet+*YrA*	unkmown	4
Avocet+*YrCV*	2AL	0
Avocet+*YrSp*	2B	0
Pavon 76 (*Yr6*, *Yr7*, *Yr29*, *Yr30*, +)	1B, 2B, 7B	1
PBW 343 (*Yr9*, *Yr27*, *Yr29*, +)	1B, 2B	4
Seri 82 (*Yr2*, *Yr9*, *Yr29*, *Yr30*, +)	1R/1B, 1B, 3B, 7B	3
Super Kauz (*Yr9*, *Yr27*, *Yr18*, *Yr30*, +)	1R/1B, 2B, 3B, 7D	3
Chuanmai45	-	0
SW1862	-	0
CN19 (*Yr41*)	2B	4
Chuanmai42 (*YrCH42=Yr26*)	1B	4

**Table 2:** Genetic analysis of seedling resistance to stripe rust pathotype No. 09-6-16-3.

Materials	No. of Plants	Infection Type (R)			Infection Type (S)			Expected Ratio	Chi-Square Test (χ^2^)	*p* Value *
		0	0	1	2	3	4			
CH45	10	10	-	-	-	-	-	-	-	-
CH42	10	-	-	-	-	-	10	-	-	-
CN19	10	-	-	-	-	-	10	-	-	-
CH45/CH42F_1_	10	-	-	-	-	-	10	-	-	-
CH45/CH42F_2_	192	13	48	23	29	18	61	7:9	0	1
CH45/CN19F_1_	10	-	-	-	-	8	2	-	-	-
CH45/CN19F_2_	206	25	43	28	9	5	96	7:9	0.7	0.4

*: *p* values were calculated from χ^2^ statistic and show significance at *p* > 0.05.

**Table 3:** Co-segregation of chromosome 1RS with rust resistance of F_2_ plants from the cross of CH45/CH42 tested against stripe rust pathotype No. 09-6-16-3.

No Plant of Homozygous 1RS		No Plant of Non Homozygous 1RS		Expected Ratio	Chi-Square Test (χ^2^)	*p* Values *
44		148		1:3	0.4	0.50
Resistance	Susceptible	Resistance	Susceptible	-	-	-
44	0	-	-	-	-	-
-	-	40	108	1:3	0.32	0.57

*: *p* values were calculated from χ^2^ statistic and show significance at *p* > 0.05.
